# Immune cell engagers in lung cancer

**DOI:** 10.3389/fimmu.2026.1746093

**Published:** 2026-04-10

**Authors:** Jianhong Kang, Mei Zhang, Ya He, Junke Chen, Xianglan Liu, Qin Lv

**Affiliations:** 1 Department of Thoracic Surgery, Sichuan Provincial People’s Hospital, University of Electronic Science and Technology of China, Chengdu, Sichuan, China; 2 Department of Pulmonary and Critical Care Medicine, Sichuan Provincial People’s Hospital, University of Electronic Science and Technology of China, Chengdu, Sichuan, China

**Keywords:** bispecific antibodies, cancer treatment, immune cell engagers, immunotherapy, lung cancer

## Abstract

In recent years, significant progress has been made in lung cancer treatment paradigms with the continuous unraveling of the tumor microenvironment and the ongoing advancement of immunotherapy. As an emerging immunotherapy modality, Immune Cell Engagers (ICEs) aim to effectively mobilize the body’s antitumor immune response by targeting tumors and activating immune effector cells such as T cells, NK cells, and myeloid cells. Recent studies have indicated that T-cell engagers (TCEs), exemplified by bispecific T-cell engagers (BiTEs), can enhance T-cell immunological activity within the lung cancer microenvironment and demonstrate significant antitumor effects in both *in vitro* and *in vivo* experiments. However, the highly heterogeneous nature of the lung cancer microenvironment and its complex immunosuppressive networks limit the therapeutic efficacy of ICEs. Meanwhile, key challenges remain in improving target cell specificity, lowering toxicity to non-target cells, and optimizing pharmacokinetics. This review systematically summarizes the mechanisms of action and recent advances of ICEs in lung cancer immunotherapy, explores innovative development directions for next-generation ICEs, and highlights their significant potential in driving paradigm shifts in lung cancer immunotherapy.

## 1 Introduction

Lung cancer refers to malignant tumors originating from bronchial epithelium or alveolar epithelium, ranking among the most burdensome malignant tumors globally. It is the second most common cancer type worldwide, with over 2.3 million new cases and more than 2 million deaths reported globally in 2023 (1). In the United States, lung cancer remains the leading cause of cancer-related deaths, accounting for approximately 25% of all cancer fatalities (2). Lung cancer can be classified into small-cell lung cancer (SCLC) and non-small-cell lung cancer (NSCLC) according to histopathological characteristics. NSCLC accounts for approximately 85% of all lung cancer cases, while SCLC constitutes the remaining 15% (3). Overall, lung cancer continues to exhibit high incidence, high mortality, and low overall survival rates globally, posing a significant public health challenge.

Over the past decade, the rapid advancement of tumor immunotherapy has profoundly transformed the treatment landscape for advanced lung cancer (4). In particular, the clinical application of immune checkpoint inhibitors (ICIs) targeting the programmed cell death protein 1 (PD-1) and its ligand (PD-L1) axis has significantly lowered the mortality risk in patients with advanced NSCLC (5–7). However, although some patients have achieved unprecedented durable responses and survival benefits, the overall response rate to ICIs remains limited. A substantial proportion of patients fail to derive clinical benefit from anti-PD-1/PD-L1 therapy (8, 9). This phenomenon mainly stems from the high heterogeneity of the lung cancer tumor microenvironment (TME). Tumor cells can downregulate major histocompatibility complex class I (MHC-I) molecules, thereby diminishing antigen presentation capacity and impeding the recognition and infiltration of effector T cells, resulting in the so-called “cold tumor” phenotype (10). This immunosuppressive microenvironment has weakened antitumor immune responses, thereby limiting the intensity and persistence of responses to ICIs and posing a key barrier to achieving widespread clinical benefit.

In order to overcome the therapeutic limitations posed by “immunologically cold tumors,” researchers are exploring novel immunotherapy strategies that can broaden current indications and activate effector immune cells. Against this backdrop, immune cell engagers (ICEs) represent one of the most significant advances. For ICEs, engineered antibody structures are utilized to achieve bispecific or multispecific binding between tumor cell antigens and receptors on immune effector cells (particularly T cells or NK cells), forming functional immune synapses between them (11, 12). Unlike ICIs that rely on pre−existing tumor−reactive T cells within the tumor microenvironment, ICEs mainly recruit immune cells from the peripheral circulation to trigger anti−tumor immune responses (13, 14). Consequently, they offer novel therapeutic possibilities for lung cancer patients who are unresponsive or resistant to ICIs. For example, the DLL3×CD3 T-cell engager tarlatamab has demonstrated significant efficacy in SCLC patients who have undergone chemotherapy and immunotherapy, leading to its FDA approval for treating recurrent extensive-stage small cell lung cancer (15, 16).

Therefore, this work systematically elucidates the molecular basis and primary types of ICEs, reviews their research progress in lung cancer immunotherapy, and further explores their future development directions in lung cancer treatment. This aims to provide reference for the research, development, and application of next-generation immune cell engagers.

## 2 Overview of ICE

### 2.1 General structure of ICE

ICEs are a class of engineered molecules based on bispecific antibodies (BsAbs) or multispecific antibodies (MsAbs). Based on their design principles and structural characteristics, different types of bispecific and multispecific antibodies exhibit variations in antigen recognition arm construction, molecular spatial conformation, and effector function regulation.

**Figure 1:**
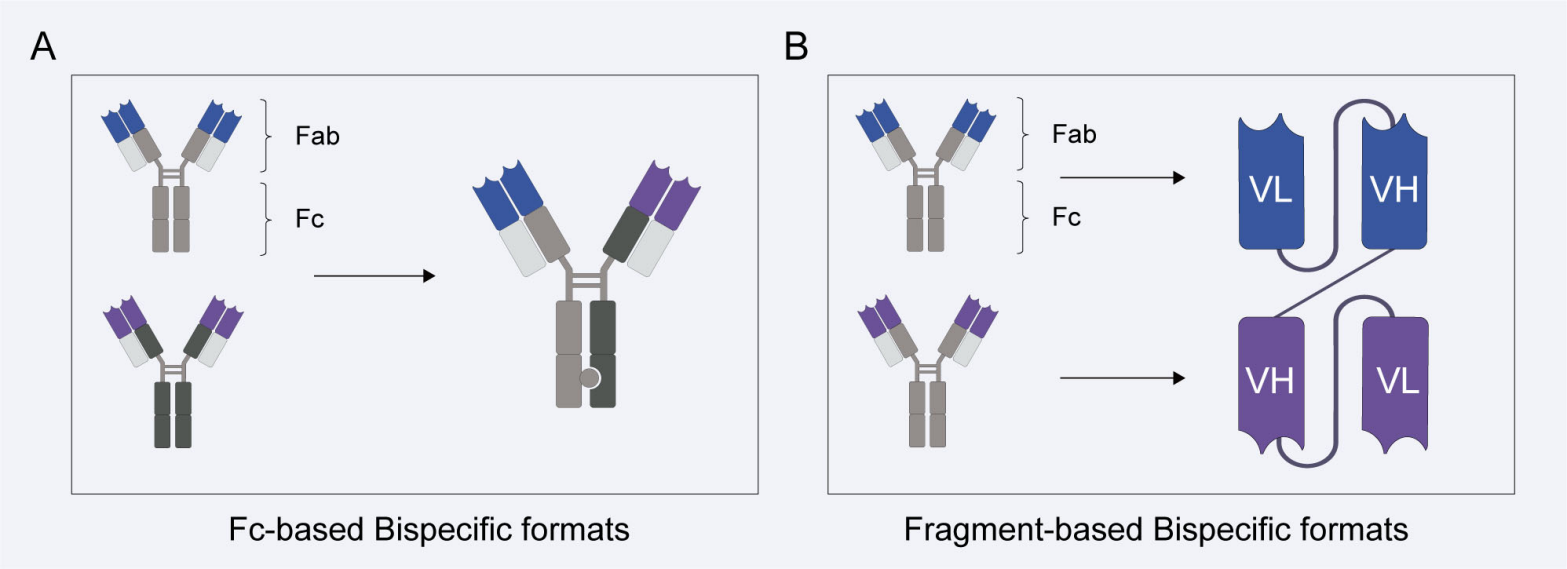
Molecular basis of immune cell engagers. **(A)** Non-IgG-like bsAbs: constructed by connecting the variable domains of IgG heavy and light chains via flexible peptide linkers. **(B)** IgG-like bsAbs: formed by fusing two distinct Fab fragments to the Fc region of an IgG-like molecule.

BsAbs is classified into two categories based on whether they contain an Fc region: Fc based bsAbs (also termed IgG-like bsAbs) and Fragment-based bsAbs (also termed non-IgG-like bsAbs) (**Figure 1**) (17). IgG-like bsAbs structurally resemble full-length IgG, containing two Fab fragments with distinct antigen specificities within the IgG molecular framework while retaining the Fc domain. Due to Fc domain, IgG-like bsAbs can trigger Fc-mediated effector functions, including antibody-dependent cellular cytotoxicity (ADCC) and complement-dependent cytotoxicity (CDC). Simultaneously, as IgG-based molecules, these antibodies exhibit higher stability, improved plasma solubility, stronger antigen affinity, and longer *in vivo* half-lives. However, their lower renal clearance may impact dosing and toxicity profiles. Non-IgG-like bsAbs are formed by fusing the variable domains of IgG heavy and light chains via flexible peptide bonds (18). Compared to IgG-like bsAbs, non-IgG-like bsAbs (11–50 kDa) exhibit enhanced tissue permeability due to their compact structure and absence of an Fc region, enabling faster renal clearance and reducing the risk of non-specific immune activation. Their activity relies solely on intrinsic antigen-binding capacity (18). However, these molecules exhibit poor solubility and stability, along with a short *in vivo* half-life, necessitating further engineering modifications to improve their pharmacokinetic properties. These biological differences among bispecific antibodies may influence their clinical development trajectories and ultimately impact their application in clinical practice.

MsAbs can simultaneously recognize multiple tumor-associated or immune-regulatory antigens, thereby demonstrating unique advantages in enhancing tumor selectivity, improving immune effector functions, and lowering off-target risks. Among these, trispecific antibodies (TsAbs) represent a quintessential example of multispecific antibodies. By adding a third binding arm to the traditional bispecific framework, TsAbs can target additional tumor-associated antigens (TAAs) to enhance specificity and reduce immune escape, or bind co-stimulatory receptors on immune cells to further amplify immune activation and antitumor effects (19). Thus, the incorporation of binding domains endows ICEs with novel and attractive properties.

### 2.2 ICE

**Figure 2:**
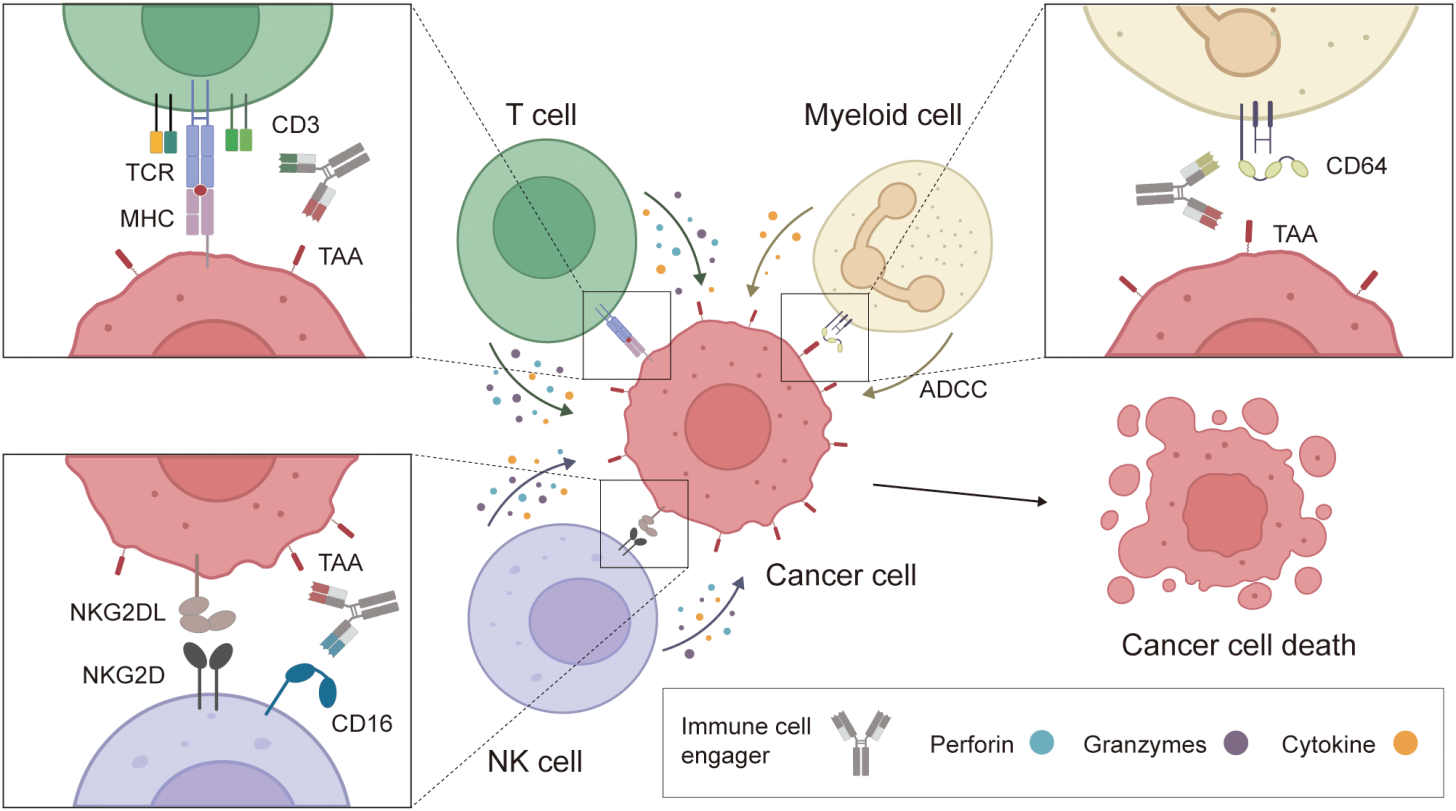
Mechanism of action of bsAb-based ICEs in cancer. The schematic illustrates bsAb-based ICEs, including T-cell, NK cell, and myeloid cell engagers, which simultaneously bind tumor-associated antigens on cancer cells and specific antigens on immune cells. These interactions promote the formation of an immunological synapse and activation of immune effector cells, leading to the release of cytokines, perforin, and granzymes that induce cytotoxic effects against tumor cells.

To date, ICEs can be categorized into three major classes: T-cell engagers, natural killer (NK) cell engagers, and myeloid cell engagers (**Figure 2**).

T-cell engagers: T-cell engagers (TCEs) represent a novel class of engineered bsAbs, primarily exemplified by bispecific T-cell engagers (BiTes). These agents achieve precise bridging between T cells and tumor cells by linking a single-chain variable fragment (scFv) targeting a tumor-associated antigen with another scFv recognizing the CD3ϵ chain of the T-cell receptor (TCR) complex (20, 21). Leveraging this structure, T cell engagers form an “artificial immune synapse” (22) between T cells and tumor cells, strongly activating signaling pathways associated with the TCR complex. Activated T cells subsequently release perforin to form pores in the tumor cell membrane and granzymes to hydrolyze cellular proteins, thereby inducing potent contact-dependent cytotoxic responses (23, 24). Unlike traditional immune effects dependent on tumor MHC molecule antigen presentation, T cell engagers function independently of MHC expression, thereby overcoming immune escape caused by MHC downregulation (25, 26). This characteristic confers unique advantages for treating “cold tumors” or tumors with low immunogenicity. CD3-complex-specific BiTes not only effectively activate CD8+ cytotoxic T cells but also recruit CD4+ T cells, γδ T cells, and NKT cells, thereby amplifying the overall antitumor immune response (27–29). The first T-cell engager drug, blinatumomab, has demonstrated potent therapeutic potential in malignant tumors, particularly hematologic malignancies expressing tumor-associated antigens (TAAs) (30). Dozens of bispecific antibody (bsAb)-based T-cell engagers are currently in clinical trials, offering new hope for treating solid tumors such as lung cancer.

NK cell engagers: NK cell engagers (NKCEs) can significantly improve tumor treatment efficacy by activating the immune function of natural killer (NK) cells (31, 32). NK cells surface harbors multiple key activation receptors, including C-type lectin receptors (e.g., CD94/NKG2C and NKG2D), natural killer cell receptors (e.g., NKp30 and NKp46), and killer cell C-type lectin-like receptors (e.g., KLRG1). Among these, CD16a, NKp46, NKp30, NKG2C, and NKG2D serve as primary targets for NKCE-based immunotherapies (33). CD16a, the major activating Fc receptor on NK cells, is also the core target for developing bispecific antibody-based NKCEs (34). CD16a-targeted NKCEs simultaneously bind to tumor-associated antigens and CD16a molecules on NK cell surfaces, effectively recruiting and activating NK cells. This induces antibody-dependent cellular cytotoxicity, leading to tumor cell lysis (35, 36). Notably, NKCE activation does not require MHC or TCR signaling, enabling efficacy even in tumors with downregulated MHC expression and low immunogenicity (37). Furthermore, compared to T cell engagers, NKCEs and TriKEs offer advantages of lower cost, reduced toxicity, and shorter production cycles (33, 38). This novel class of immunotherapies provides a significant avenue for overcoming immune suppression in complex tumor microenvironments and targeting solid tumors.

Myeloid Cell Engagers: Myeloid cells are highly enriched in the tumor microenvironment and exhibit significant plasticity (39, 40), endowing myeloid cell engagers (MCEs) with broad application potential in both “hot tumors” and “cold tumors.” MCEs can trigger Fc receptor (FcR)-mediated antibody-dependent cellular phagocytosis in bone marrow cells or activate myeloid cells by targeting immunomodulatory or immunostimulatory molecules, thereby enhancing antigen-presenting cell function (41, 42). For example, bispecific macrophage engagers (BiME) simultaneously bind tumor-associated antigens on cancer cells and activation receptors on macrophages, such as Fcγ receptors (FcγRI, FcγRIIa, and FcγRIIIa), inducing antibody-dependent cellular phagocytosis (ADCP) and cytokine secretion (43). Simultaneously, MCEs can function as antigen-independent therapeutics that modulate the tumor microenvironment, enhancing immune responses by reshaping immunosuppressive conditions. For instance, bispecific dendritic cell–T cell engagers (BiCEs) enhance antitumor immunity by targeting dendritic cells (DCs). Upon binding to CLEC9A, it promotes the migration and antigen-presenting functions of cDC1, enhances the formation of immune synapses between PD-1^+^ T cells and DCs, and induces upregulation of IL-12 secretion, thereby driving T cell-mediated antitumor immune responses (44).

T−cell engagers, NK−cell engagers, and myeloid cell engagers represent three major immune cell–engaging strategies. Although these therapies elicit potent antitumor immune responses, they may also cause immune−related toxicities. For instance, overactivation of T cells by ICEs can rapidly trigger the release of large amounts of pro−inflammatory cytokines, which further activate monocytes and macrophages, amplifying inflammatory cascades and leading to cytokine release syndrome (CRS) (45). Moreover, excessive cytokines and immune−mediated endothelial activation can increase blood–brain barrier permeability, resulting in immune effector cell–associated neurotoxicity syndrome (ICANS) (46). Therefore, minimizing toxicity while maintaining or enhancing antitumor efficacy has become a key focus in ICEs development. With advances in antibody engineering and a deeper understanding of tumor−associated targets, next−generation ICEs are expected to achieve a better balance between efficacy and safety, facilitating their clinical translation in cancer therapy.

## 3 Research and application advances of ICEs in lung cancer

The advent of immunotherapy has provided new therapeutic opportunities for lung cancer. Immunotherapy combined with chemotherapy has been established as the first−line treatment for extensive−stage small cell lung cancer (ES-SCLC). ICEs have emerged as a novel immunotherapeutic strategy in lung cancer. In 2025, the DLL3 × CD3 bispecific T−cell engager tarlatamab was approved in the United States for the treatment of small cell lung cancer, marking the clinical translation of ICEs. Meanwhile, multiple ICEs have been evaluated in preclinical studies and clinical trials. Here, we summarize recent advances of ICEs in lung cancer (**Table 1**).

**Table 1:** Summary of efficacy data from trials testing immune cell engagers for lung cancers.

Name	Design	Target	Indication	NCT	Phase	Indication	Key outcomes	Ref.
Tarlatamab	T cell engager	DLL × CD3	relapsed/refractory SCLC	NCT03319940	1	Active	Monotherpy (n = 107): ORR 23.4%, mDOR 12.3 months, DCR 51.4%, mPFS 3.7 months, and mOS 13.2 months.	(15)
			Previously treated SCLC	NCT05060016	2	Active	Monotherapy (n = 220): ORR 32–40%, mPFS 3.9–4.9 months, and 9-month OS 66–68%	(16)
			After Platinum-Based Chemotherapy SCLC	NCT05740566	3	Active	Monotherapy (n = 168): mOS 13.6 vs. 8.3 months	(51)
			ES-SCLC	NCT06211036	1	Active	Monotherapy with atezolizumab or durvalumab ((n = 88): ORR 24%, mPFS 5.6 months, mOS 25.3 months, DCR 60%, and mDOR 16.6 months.	(98)
Obrixtamig	T cell engager	DLL × CD3	Previously treated SCLC, epNEC, LCNEC-L	NCT04429087	1	Recruiting	Monotherapy (n = 509): ORR 23%, mDOR 8.5 months, and 6-month DOR 70%.	(54)
			Advanced SCLC	NCT05990738	1	Recruiting	Monotherapy and with topotecan ((n = 25): unconfirmed ORR 70% (1 CR, 15 PR) and DCR 87%.	(55)
AFM24	NK cell engager	CD16A × EGFR	Previously treated SCLC	NCT05109442	2	Terminated	AFM24 plus atezolizumab: ORR 23% among 35 evaluable patients and DCR 77%.	(60)
Gocatamig	Trispecific T cell engager	DLL3 × HAS × CD3	Including relapsed/refractory, metastatic SCLC	NCT04471727.	1/2	Recruiting	Monotherapy: Confirmed ORR 50% among 24 SCLC patients	(65)
ZG006	Trispecific T cell engager	DLL3 × DLL3× CD3	Refractory SCLC or NEC	NCT05978284.	1	Recruiting	Monotherapy: ORR 60.9% among 23 evaluable SCLC patients and DCR 78.3%.	(66)
			Advanced SCLC	NCT06283719	2	Recruiting	Monotherapy(n = 60) : ORR 53.8–78.6% and DCR 92.6–100%	(67)
RG-6524	Trispecific T cell engager	DLL3 × CD137× CD3	Including advanced SCLC	NCT07107490	1	Not yet recruiting	\	

Tarlatamab (AMG757): DLL3 is highly expressed on the surface of approximately 85%–96% of SCLC cells, while exhibiting extremely low expression in normal tissues, making it a potential therapeutic target for SCLC (47, 48). Thus, Giffin et al. developed an immunotherapy strategy targeting DLL3—Tarlatamab (AMG 757)—a half-life-extended anti-DLL3 × anti-CD3 bispecific T cell-engaging antibody (49). It binds to DLL3 on cancer cells and CD3 on T cells, forming a cytolytic synapse that activates T cells independently of MHC class I, leading to T cell-mediated cancer cell lysis (50). Tarlatamab efficacy was evaluated in SCLC cell lines and in SCLC mouse models using both *in situ* and patient-derived xenografts (PDX). Tarlatamab effectively engages T cells, induces T cell activation, and directs T cells to lyse tumor cells, demonstrating potent and specific killing effects even against SCLC cell lines with extremely low DLL3 expression (fewer than 1000 molecules per cell surface). It significantly promoted tumor regression in both PDX SCLC models and orthotopic models (49). In non-human primates (NHPs), Tarlatamab demonstrated good tolerability and an extended half-life (49). Tarlatamab exhibited favorable safety and significant clinical benefit in the Phase I DeLLphi-300 trial (NCT03319940) for recurrent or refractory SCLC. The study enrolled SCLC patients who had received prior multi-line therapy, yielding an ORR of 23.4%, a median PFS of 3.7 months, and an OS of 13.2 months (15). Although over half of patients experienced CRS, severe toxicity (≥ Grade 3) was uncommon, and all events were reversible without treatment discontinuation (15). The Phase II DeLLphi-301 (NCT05060016) study has been performed to compare the efficacy of tarlatamab administered intravenously every 2 weeks at two dose levels (10 mg and 100 mg). The results demonstrated comparable ORR (40% vs. 32%) and median PFS (4.9 months vs. 3.9 months) between 10 mg and 100 mg tarlatamab administered intravenously every 2 weeks, with lower treatment-related adverse event rates (16). In November 2025, tarlatamab-dlle (IMDELLTRA) was approved as the first FDA-authorized DLL3-targeted bispecific T cell engager therapy for adult patients with ES-SCLC whose disease progressed during or after platinum-based chemotherapy (51). It has been reported that tarlatamab significantly improves overall survival (OS) in patients with platinum-resistant disease compared to standard chemotherapy (13.6 months vs. 8.3 months), along with advantages in progression-free survival and cancer-related symptom relief, while exhibiting a lower incidence of serious adverse events (NCT05740566) (54% vs. 80%) (52).

Obrixtamig (BI764532): Obrixtamig is an IgG-like bispecific T-cell engager targeting DLL3 and CD3. In SCLC cell lines and primary human effector cells, Obrixtamig selectively binds DLL3-positive tumor cells and T cells, inducing the formation of immune synapses that lead to tumor cell lysis and T cell activation. In human T-cell-transplanted xenograft models, Obrixtamig enhances T-cell infiltration, induces tumor cell apoptosis, and results in significant tumor regression. Non-human primate studies indicate a half-life of approximately 10 days, and its IgG-like structure may confer lower immunogenicity risk compared to Tarlatamab (53). In Phase I clinical trials (NCT04429087), Obrixtamig demonstrated favorable safety and notable antitumor activity in patients with DLL3-positive SCLC, epNEC, and LCNEC-L who had received multiple prior lines of therapy. Among 168 patients receiving four distinct dosing regimens, no maximum tolerated dose (MTD) was observed. The most common related adverse event was cytokine release syndrome (CRS; 57%, ≥Grade 3 in 3%), predominantly early-onset and reversible. The overall objective response rate (ORR) reached 23%, with a median response duration of 8.5 months and a 6-month sustained response rate of 70%. In the effective dose group (≥90 μg/kg), ORR increased to 28%, with ORRs of 21%, 27%, and 70% for SCLC, epNEC, and LCNEC-L patients, respectively (54). Interim results from the latest Phase Ib study (NCT05990738) evaluating obrixtamig in combination with topotecan for advanced SCLC patients demonstrated encouraging preliminary findings. The unconfirmed ORR was 70%, with a disease control rate of 87% and favorable safety profiles (55).

AFM24: Epidermal growth factor receptor (EGFR) is a transmembrane glycoprotein belonging to the erbB family of tyrosine kinase receptors (56). Binding of EGFR to its ligand activates multiple signaling pathways involved in regulating cell proliferation, differentiation, and survival. Although EGFR is expressed in normal cells, it is frequently overexpressed in NSCLC. This abnormal activation is closely associated with tumor progression, poor prognosis, and reduced survival rates (57). Targeting this molecular signature, Affirmed developed AFM24—a bispecific natural killer cell engager that simultaneously targets EGFR and CD16a. By recruiting and activating NK cells, it selectively mediates immune-mediated killing of EGFR-positive tumor cells (58). AFM24 functions through immune cell-mediated killing instead of EGFR signaling inhibition, maintaining activity in tumors with EGFR or downstream pathway mutations while reducing toxicities associated with traditional EGFR inhibitors. AFM24 binds CD16A and EGFR with high affinity at low nanomolar levels, inducing NK cell-mediated ADCC and macrophage-mediated ADCP, demonstrating significant antitumor activity. *In vivo* studies indicate good tolerability (maximum dose 75 mg/kg) with a half-life of approximately 28 days in non-human primates (58). A Phase I clinical trial enrolled 35 patients with advanced EGFR-positive solid tumors, demonstrating the overall safety and tolerability of AFM24, with infusion-related reactions as the most common adverse events. A dose of 480 mg/week was established as the recommended Phase II dose (59). In a Phase 2a trial (AFM24-102, NCT05109442) evaluating AFM24 in combination with atezolizumab for advanced/metastatic NSCLC patients with EGFR kinase domain mutations, preliminary results from 22 evaluable patients showed an ORR of 23% (1 CR, 3 PR, and 1 unconfirmed PR), a DCR of 64%, and tumor shrinkage in 50% of patients. The preliminary median PFS was 5.5 months. The most common AFM24-related adverse events were infusion-related reactions (65%) (60). Based on these findings, the FDA granted Fast Track designation to the combination of AFM24 and atezolizumab (Tecentriq) for the treatment of patients with advanced and/or metastatic NSCLC harboring no EGFR mutation who have progressed after immunotherapy and platinum-based chemotherapy.

Gocatamig (HPN328): HPN328 is a half-life-extended trispecific T cell engager consisting of three binding domains: a CD3 binder for T-cell engagement, an albumin binder for half-life extension, and a DLL3 binder for tumor cell engagement (61). Concurrently, the compact molecular size design of the trispecific structure enhances drug penetration into solid tumors, overcoming the steric hindrance limitations of traditional bispecific antibodies (62, 63). HPN328 specifically binds DLL3 and directs T cells to kill tumor cells, demonstrating significant antitumor activity *in vitro* and in mouse xenograft models. In cynomolgus monkey studies, HPN328 exhibited good tolerability and favorable pharmacokinetic properties, with a serum half-life ranging from 78 to 187 hours (64). A Phase 1/2 trial (NCT04471727) evaluating HPN328 monotherapy in advanced DLL3+ malignancies, including ES-SCLC, reported updated results: in the evaluable cohort, the confirmed ORR in SCLC was 50% (12/24), with 1 complete response (65). These findings underscore its potential as a next-generation TCE for solid tumors.

ZG006: ZG006 is a trispecific T cell engager targeting DLL3 and CD3. By simultaneously binding to two distinct DLL3 epitopes on tumor cells and CD3 on T cells, it bridges and activates T cells to kill DLL3-positive tumor cells (66). Results from a Phase I trial (NCT05978284) results demonstrated the favorable safety profile of ZG006 and significant antitumor activity in patients with SCLC and NEC who had failed prior standard therapies, achieving an ORR of 60.9%. A 66.7% response rate was maintained even in the low/intermediate DLL3 expression cohort. Primary adverse events included mild-to-moderate cytokine release syndrome and fever (66). Phase II studies further validated ZG006 efficacy in previously treated SCLC (NCT06283719) and NEC (NCT06440057) patients: the SCLC cohort achieved an overall ORR of 66.7% (78.6% in the 30 mg group) and a disease control rate (DCR) of 92.6%. Preliminary NEC cohort data showed an ORR of 33.3% and DCR of 66.7% (67). In fact, most adverse events were manageable without treatment-related deaths. Overall, ZG006 continues to demonstrate significant activity in solid tumors with low DLL3 expression, exhibiting a favorable safety profile and potential for further clinical development.

RG-6524: CD137 is a member of the tumor necrosis factor receptor superfamily that promotes T cell proliferation, survival, maintenance, and activation (68, 69). Studies indicate that CD137 agonists can enhance the antitumor effects of bispecific T cell engagers by increasing effector T cell numbers and prolonging T cell activation (70, 71). Therefore, inducing concurrent CD137 co-stimulation may amplify the efficacy of T cell engagers, offering a promising therapeutic approach for SCLC patients. To integrate CD137 co-stimulatory function into T cell engager formats for enhanced therapeutic efficacy, Mikami et al. generated a DLL3/CD3/CD137 trispecific antibody. This molecular configuration enables competitive binding to both CD3 and CD137, thereby avoiding off-target DLL3 cross-linking while further promoting efficient activation of tumor-specific T cells. Compared to conventional DLL3-targeting bispecific T cell engagers, the DLL3/CD3/CD137 trispecific antibody induced a significant increase in T cell numbers within tumors and improved tumor growth inhibition in SCLC models (72). Hence, a Phase I trial (NCT07107490) was conducted to evaluate the safety and efficacy of ALPS12 (RO7616789) in patients with ES-SCLC.

Nb-TriTE: Human leukocyte antigen-G (HLA-G) is an immune checkpoint (ICP) molecule and novel tumor-associated antigen. Studies indicate its potential synergistic benefits as a co-target with anti-PD-L1 therapy (73, 74). Based on this, Lin et al. developed Nb-TriTE, a nanobody (Nb)-based dual immune checkpoint (ICP) targeting T cell engager composed of anti-PD-L1, anti-HLA-G, and anti-CD3 Nbs (75). Nb-TriTE demonstrated broad-spectrum antitumor activity *in vitro* by enhancing human peripheral blood mononuclear cell (PBMC)-mediated cytotoxicity. In humanized immunodeficient mouse models of NSCLC, Nb-TriTE exhibited superior anticancer efficacy compared to monoclonal antibodies and bispecific T cell engagers. At pharmacologically effective doses, Nb-TriTE did not induce additional enhancement of circulating cytokine secretion by PBMCs. Nb-TriTE effectively prolonged mouse survival without significant adverse events (75). Future research on Nb-TriTE is expected to advance further into clinical phases.

Overall, ICEs share the common goal of redirecting immune effector cells to tumor cells, but they differ in molecular design and physicochemical properties. For instance, both tarlatamab and obrixtamig target DLL3 and recruit CD3^+^ T cells. Tarlatamab incorporates a half−life extension design that enables sustained *in vivo* exposure and less frequent dosing, whereas obrixtamig adopts an IgG−like structure that may provide greater stability and lower immunogenicity. Although clinical studies highlight the therapeutic potential of ICEs in lung cancer, their limitations remain to be fully defined. By recruiting peripheral immune cells, ICEs may partially overcome insufficient immune infiltration in tumors; however, whether prolonged treatment induces peripheral immune cell exhaustion and affects subsequent therapies warrants further investigation.

## 4 Innovative strategies for ICEs in lung cancer

In recent years, ICEs have garnered significant attention for their application in lung cancer treatment. Although multiple clinical studies have demonstrated their potential advantages in activating the immune system and specifically eliminating tumor cells, numerous limitations remain to be overcome. Current major challenges include short *in vivo* half-life, off-target toxicity, limited efficacy due to immunosuppressive microenvironments, and insufficient activation of anti-tumor immunity caused by single-target limitations. To address these challenges, current research focuses on the following directions.

### 4.1 Half-life extended ICE

Half-Life Extended ICE: Molecular structure optimization is the key to enhance ICE half-life. By modulating molecular conformation, ICE half-life *in vivo* can be extended (76). Several strategies have been explored to overcome this challenge, such as conjugation with engineered IgG Fc domains, human serum albumin, or polyethylene glycol (77–79). For instance, half-life extended BiTE (HLE-BiTE) incorporates an Fc domain into the BiTE molecule, thereby sustaining serum concentrations and prolonging serum half-life, enabling once-weekly dosing in patients (80). AMG 757 is an HLE-BiTE developed based on this design principle, achieving prolonged circulation time through fusion of a non-functional Fc domain and demonstrating significantly extended half-life in non-human primates (49). However, introducing ancillary structures into ICE inevitably increases its molecular size, hindering tissue penetration and potentially elevating immunogenicity (81). Therefore, designing half-life-extended ICE requires balancing prolonged circulation time with maintaining favorable tissue permeability and low immunogenicity to achieve optimal therapeutic outcomes.

### 4.2 Enhancing tumor specificity in lung cancer

In hematologic malignancies, there is typically reversible ICE-induced depletion of B cells and myeloid cells. However, in solid tumor therapy, this targeted effect may cause severe organ damage or even death due to lower tolerance in normal tissues (82, 83). Currently, most ICEs target tumor-associated antigens, lacking true tumor-specific antigens (TSAs), thereby increasing the risk of off-tumor toxicity (84). Ideal TAA targets are antigens exclusively expressed by lung cancer cells, critical for tumor growth, and low or absent in normal cells (especially normal lung tissue). This avoids selecting antigens expressed in multiple normal organs, thereby reducing “off-target” toxicity. Examples include carcinoembryonic antigen (CEA) in non-small cell lung cancer (85), and DLL3, prostate stem cell antigen (PSCA), prostate-specific membrane antigen (PSMA), and SSTR2 in small cell lung cancer (86–89). Additionally, targeting peptide-MHC complexes to enhance ICE tumor selectivity represents an innovative strategy. Peptide-MHC complexes (pMHCs) represent cancer-specific epitopes presented on the surface of tumor cells via MHC class I molecules to T lymphocytes (90). Engineered soluble TCRs and their derivatives can bind to these pMHC complexes with high specificity. ImmTAC is an engineered TCR technology that functions by targeting HLA-peptide complexes on cancer cell surfaces. Brenetafusp, an ImmTAC targeting the preferentially expressed melanoma antigen (PRAME), is currently being evaluated in a Phase I-II clinical trial (NCT04262466) for patients with PRAME-positive advanced solid tumors. Notably, PRAME is expressed in about 20-40% of NSCLC cases (91). Concurrently, increasing the number of binding sites between antibodies and tumor antigens can enhance targeting specificity. A representative example is the bispecific T-cell engager cibisatamab (RO6958688) (92), which contains two Fab domains targeting carcinoembryonic antigen (CEA) and one Fab domain targeting CD3. This molecule is currently in Phase I clinical trials for the treatment of locally advanced or metastatic CEA-positive solid tumors (93).

### 4.3 Targeting co-stimulatory receptors

Co-stimulatory signaling pathways play a central role in T cell activation, proliferation, differentiation, and effector functions. To overcome the limited efficacy of traditional BiTE therapies due to co-stimulatory deficiency, researchers proposed the Simultaneous Multiple Interaction T-cell Engager (SMITE) strategy. This approach achieves synergistic effects by combining multiple BiTE molecules, each targeting a tumor-associated antigen while binding to either CD3 or CD28, thereby delivering both T cell activation and co-stimulatory signals. Notably, within the PD-L1×CD28 BiTE-containing SMITE system, PD-L1 signaling no longer suppresses T cell function but instead induces positive co-stimulatory activation via CD28. These activated T cells subsequently mediate efficient tumor cell killing via a second TAA×CD3 BiTE (94). Additionally, another innovative approach involves integrating co-stimulatory receptor signals such as CD137 (4-1BB) or CD28 through multivalent or multispecific ICE to enhance sustained activation and anti-tumor responses in effector T cells (95). For example, RG-6524, a trispecific antibody targeting CD137, effectively suppressed tumor growth in SCLC models by promoting efficient T cell activation while providing tumor-specific recognition (72). Collectively, these innovative ICE strategies centered on co-stimulatory receptors enhance T cell activation and antitumor effects, offering novel approaches to improve the efficacy of lung cancer immunotherapy.

### 4.4 Combination strategies for ICP inhibitors

Upregulation of inhibitory immune checkpoints is considered a key mechanism underlying resistance to ICE therapy. Preclinical studies demonstrate that combining ICE therapy with PD-1/PD-L1 pathway blockade significantly enhances ICE’s antitumor effects (96). Although DLL3-targeted BiTE can effectively activate T cells, SCLC tumors can still achieve immune escape by upregulating the PD-1/PD-L1 pathway (53). Chen et al. demonstrated that combining DLL3-BiTE with PD-1 inhibitors significantly enhanced antitumor activity *in vivo* compared to monotherapy, further validating this strategy’s potential advantage in overcoming adaptive immune tolerance (97). Furthermore, recent clinical studies suggest that tarlatamab combined with PD-L1 inhibitors demonstrates favorable safety and significant antitumor activity in maintenance therapy following first-line treatment for extensive-stage SCLC, offering a new direction for combination immunotherapy strategies in SCLC (98). Collectively, the combination of ICE agents with ICP inhibitors holds promise for enhancing immune responses and delaying the onset of resistance, providing novel therapeutic approaches for lung cancer immunotherapy.

## 5 Discussion

In recent years, ICEs have been a focal point in both research and clinical settings. Multiple innovative therapeutic strategies have demonstrated ideal efficacy outcomes in preclinical studies and early-phase clinical trials for lung cancer (52, 54, 58, 64, 75). Notably, Tarlatamab received accelerated approval from the U.S. FDA in 2024 for second-line treatment of adult patients with ES-SCLC (51). This milestone advancement signifies a substantial breakthrough for ICE-based therapies in lung cancer immunotherapy. However, due to the complex tumor microenvironment of lung cancer, there are numerous challenges in the application of ICEs in lung cancer treatment, including off-target toxicity, drug resistance, and limited efficacy (41, 99). Therefore, developing next-generation immune cell engagers for lung cancer has become a critical future research direction. Current preclinical and clinical studies are focused on optimizing ICE structural design to enhance targeting precision and antitumor efficacy. For instance, next-generation antibodies represented by multispecific ICE can effectively overcome antigen escape issues associated with single-target strategies by simultaneously recognizing multiple tumor-associated antigens or binding different effector cell types (100). Additionally, structural innovations, such as extended half-lives, improved linker designs, and the incorporation of modifiable elements can enhance ICE pharmacokinetics and reduce immunogenicity, thereby enabling more stable and sustained antitumor effects (76). At the same time, exploring synergistic interactions between ICEs and other innovative therapies, such as immune checkpoint inhibitors (98, 101) and chimeric antigen receptor (CAR) T-cell therapies (102, 103) may represent an ideal strategy to enhance the efficacy of immunotherapy in lung cancer.

Overall, ICE-based therapies offer a novel therapeutic approach for lung cancer immunotherapy by reconnecting tumor cells with the immune system, potentially overcoming the efficacy limitations of existing immunotherapies. As more novel ICE agents advance into clinical trials, ICE-based therapies are poised to reshape the landscape of lung cancer immunotherapy and deliver therapeutic benefits to patients.

## References

[1] Collaborators GBDC . The global, regional, and national burden of cancer, 1990-2023, with forecasts to 2050: a systematic analysis for the Global Burden of Disease Study 2023. Lancet. (2025) 406:1565–86. doi: 10.2139/ssrn.6378361. PMID: 41015051 PMC12687902

[2] Siegel RL Kratzer TB Giaquinto AN Sung H Jemal A . Cancer statistics, 2025. CA Cancer J Clin. (2025) 75:10–45. doi: 10.3322/caac.21871. PMID: 39817679 PMC11745215

[3] Ricotti A Sciannameo V Balzi W Roncadori A Canavese P Avitabile A . Incidence and prevalence analysis of non-small-cell and small-cell lung cancer using administrative data. Int J Environ Res Public Health. (2021) 18:9076. doi: 10.3390/ijerph18179076. PMID: 34501665 PMC8431612

[4] Lahiri A Maji A Potdar PD Singh N Parikh P Bisht B . Lung cancer immunotherapy: progress, pitfalls, and promises. Mol Cancer. (2023) 22:40. doi: 10.1186/s12943-023-01740-y. PMID: 36810079 PMC9942077

[5] Borghaei H Gettinger S Vokes EE Chow LQM Burgio MA de Castro Carpeno J . Five-year outcomes from the randomized, phase III trials CheckMate 017 and 057: Nivolumab versus docetaxel in previously treated non-small-cell lung cancer. J Clin Oncol. (2021) 39:723–33. doi: 10.1200/jco.20.01605. PMID: 33449799 PMC8078445

[6] Garassino MC Gadgeel S Speranza G Felip E Esteban E Domine M . Pembrolizumab plus pemetrexed and platinum in nonsquamous non-small-cell lung cancer: 5-year outcomes from the phase 3 KEYNOTE-189 study. J Clin Oncol. (2023) 41:1992–8. doi: 10.1200/jco.22.01989. PMID: 36809080 PMC10082311

[7] Novello S Kowalski DM Luft A Gumus M Vicente D Mazieres J . Pembrolizumab plus chemotherapy in squamous non-small-cell lung cancer: 5-year update of the phase III KEYNOTE-407 study. J Clin Oncol. (2023) 41:1999–2006. doi: 10.1200/jco.22.01990. PMID: 36735893 PMC10082300

[8] Foy JP Karabajakian A Ortiz-Cuaran S Boussageon M Michon L Bouaoud J . Immunologically active phenotype by gene expression profiling is associated with clinical benefit from PD-1/PD-L1 inhibitors in real-world head and neck and lung cancer patients. Eur J Cancer. (2022) 174:287–98. doi: 10.1016/j.ejca.2022.06.034. PMID: 36038492

[9] Konen JM Wu H Gibbons DL . Immune checkpoint blockade resistance in lung cancer: emerging mechanisms and therapeutic opportunities. Trends Pharmacol Sci. (2024) 45:520–36. doi: 10.1016/j.tips.2024.04.006. PMID: 38744552 PMC11189143

[10] Fuca G Spagnoletti A Ambrosini M de Braud F Di Nicola M . Immune cell engagers in solid tumors: promises and challenges of the next generation immunotherapy. ESMO Open. (2021) 6:100046. doi: 10.1016/j.esmoop.2020.100046, PMID: 33508733 PMC7841318

[11] Rui R Zhou L He S . Cancer immunotherapies: advances and bottlenecks. Front Immunol. (2023) 14:1212476. doi: 10.3389/fimmu.2023.1212476. PMID: 37691932 PMC10484345

[12] Zhou S Liu M Ren F Meng X Yu J . The landscape of bispecific T cell engager in cancer treatment. biomark Res. (2021) 9:38. doi: 10.1186/s40364-021-00294-9. PMID: 34039409 PMC8157659

[13] Tian Z Liu M Zhang Y Wang X . Bispecific T cell engagers: an emerging therapy for management of hematologic Malignancies. J Hematol Oncol. (2021) 14:75. doi: 10.1186/s13045-021-01084-4. PMID: 33941237 PMC8091790

[14] de Miguel M Umana P Gomes de Morais AL Moreno V Calvo E . T-cell-engaging therapy for solid tumors. Clin Cancer Res. (2021) 27:1595–603. doi: 10.1158/1078-0432.ccr-20-2448. PMID: 33082210

[15] Paz-Ares L Champiat S Lai WV Izumi H Govindan R Boyer M . Tarlatamab, a first-in-class DLL3-targeted bispecific T-cell engager, in recurrent small-cell lung cancer: an open-label, phase I study. J Clin Oncol. (2023) 41:2893–903. doi: 10.1200/jco.22.02823. PMID: 36689692 PMC10414718

[16] Ahn MJ Cho BC Felip E Korantzis I Ohashi K Majem M . Tarlatamab for patients with previously treated small-cell lung cancer. N Engl J Med. (2023) 389:2063–75. doi: 10.1056/nejmoa2307980. PMID: 37861218

[17] Spiess C Zhai Q Carter PJ . Alternative molecular formats and therapeutic applications for bispecific antibodies. Mol Immunol. (2015) 67:95–106. doi: 10.1016/j.molimm.2015.01.003. PMID: 25637431

[18] Labrijn AF Janmaat ML Reichert JM Parren P . Bispecific antibodies: a mechanistic review of the pipeline. Nat Rev Drug Discov. (2019) 18:585–608. doi: 10.1038/s41573-019-0028-1. PMID: 31175342

[19] Tapia-Galisteo A Compte M Alvarez-Vallina L Sanz L . When three is not a crowd: trispecific antibodies for enhanced cancer immunotherapy. Theranostics. (2023) 13:1028–41. doi: 10.7150/thno.81494. PMID: 36793863 PMC9925307

[20] Zhu M Wu B Brandl C Johnson J Wolf A Chow A . Blinatumomab, a bispecific T-cell engager (BiTE((R))) for CD-19 targeted cancer immunotherapy: clinical pharmacology and its implications. Clin Pharmacokinet. (2016) 55:1271–88. doi: 10.1007/s40262-016-0405-4. PMID: 27209293

[21] Lou H Cao X . Antibody variable region engineering for improving cancer immunotherapy. Cancer Commun (Lond). (2022) 42:804–27. doi: 10.1002/cac2.12330. PMID: 35822503 PMC9456695

[22] Kamakura D Asano R Yasunaga M . T cell bispecific antibodies: an antibody-based delivery system for inducing antitumor immunity. Pharm (Basel). (2021) 14:1172. doi: 10.3390/ph14111172. PMID: 34832954 PMC8619951

[23] Haas C Krinner E Brischwein K Hoffmann P Lutterbuse R Schlereth B . Mode of cytotoxic action of T cell-engaging BiTE antibody MT110. Immunobiology. (2009) 214:441–53. doi: 10.1016/j.imbio.2008.11.014. PMID: 19157637

[24] Thiery J Keefe D Boulant S Boucrot E Walch M Martinvalet D . Perforin pores in the endosomal membrane trigger the release of endocytosed granzyme B into the cytosol of target cells. Nat Immunol. (2011) 12:770–7. doi: 10.1038/ni.2050. PMID: 21685908 PMC3140544

[25] de Sostoa J Fajardo CA Moreno R Ramos MD Farrera-Sal M Alemany R . Targeting the tumor stroma with an oncolytic adenovirus secreting a fibroblast activation protein-targeted bispecific T-cell engager. J Immunother Cancer. (2019) 7:19. doi: 10.1186/s40425-019-0505-4. PMID: 30683154 PMC6347837

[26] Singh K Hotchkiss KM Mohan AA Reedy JL Sampson JH Khasraw M . For whom the T cells troll? Bispecific T-cell engagers in glioblastoma. J Immunother Cancer. (2021) 9:e003679. doi: 10.1136/jitc-2021-003679. PMID: 34795007 PMC8603282

[27] Quintarelli C Orlando D Boffa I Guercio M Polito VA Petretto A . Choice of costimulatory domains and of cytokines determines CAR T-cell activity in neuroblastoma. Oncoimmunology. (2018) 7:e1433518. doi: 10.1080/2162402x.2018.1433518. PMID: 29872565 PMC5980417

[28] Perez-Ruiz E Etxeberria I Rodriguez-Ruiz ME Melero I . Anti-CD137 and PD-1/PD-L1 antibodies en route toward clinical synergy. Clin Cancer Res. (2017) 23:5326–8. doi: 10.1158/1078-0432.ccr-17-1799. PMID: 28790118

[29] Nguyen HH Kim T Song SY Park S Cho HH Jung SH . Naive CD8(+) T cell derived tumor-specific cytotoxic effectors as a potential remedy for overcoming TGF-beta immunosuppression in the tumor microenvironment. Sci Rep. (2016) 6:28208. doi: 10.1038/srep28208. PMID: 27306834 PMC4910083

[30] Sanford M . Blinatumomab: first global approval. Drugs. (2015) 75:321–7. doi: 10.1007/s40265-015-0356-3. PMID: 25637301

[31] Wu SY Fu T Jiang YZ Shao ZM . Natural killer cells in cancer biology and therapy. Mol Cancer. (2020) 19:120. doi: 10.1186/s12943-020-01238-x. PMID: 32762681 PMC7409673

[32] Shin MH Oh E Minn D . Current developments in NK cell engagers for cancer immunotherapy: focus on CD16A and NKp46. Immune Netw. (2024) 24:e34. doi: 10.4110/in.2024.24.e34. PMID: 39513028 PMC11538608

[33] Liu S Galat V Galat Y Lee YKA Wainwright D Wu J . NK cell-based cancer immunotherapy: from basic biology to clinical development. J Hematol Oncol. (2021) 14:7. doi: 10.1186/s13045-020-01014-w. PMID: 33407739 PMC7788999

[34] Bryceson YT March ME Ljunggren HG Long EO . Synergy among receptors on resting NK cells for the activation of natural cytotoxicity and cytokine secretion. Blood. (2006) 107:159–66. doi: 10.1182/blood-2005-04-1351. PMID: 16150947 PMC1895346

[35] Gleason MK Verneris MR Todhunter DA Zhang B McCullar V Zhou SX . Bispecific and trispecific killer cell engagers directly activate human NK cells through CD16 signaling and induce cytotoxicity and cytokine production. Mol Cancer Ther. (2012) 11:2674–85. doi: 10.1158/1535-7163.mct-12-0692. PMID: 23075808 PMC3519950

[36] Prager I Watzl C . Mechanisms of natural killer cell-mediated cellular cytotoxicity. J Leukoc Biol. (2019) 105:1319–29. doi: 10.1002/jlb.mr0718-269r. PMID: 31107565

[37] Zhu A Bai Y Nan Y Ju D . Natural killer cell engagers: from bi-specific to tri-specific and tetra-specific engagers for enhanced cancer immunotherapy. Clin Transl Med. (2024) 14:e70046. doi: 10.1002/ctm2.70046. PMID: 39472273 PMC11521791

[38] Zhang M Lam KP Xu S . Natural killer cell engagers (NKCEs): a new frontier in cancer immunotherapy. Front Immunol. (2023) 14:1207276. doi: 10.3389/fimmu.2023.1207276. PMID: 37638058 PMC10450036

[39] Kim J Bae JS . Tumor-associated macrophages and neutrophils in tumor microenvironment. Mediators Inflammation. (2016) 2016:6058147. doi: 10.1155/2016/6058147. PMID: 26966341 PMC4757693

[40] He S Zheng L Qi C . Myeloid-derived suppressor cells (MDSCs) in the tumor microenvironment and their targeting in cancer therapy. Mol Cancer. (2025) 24:5. doi: 10.1186/s12943-024-02208-3. PMID: 39780248 PMC11707952

[41] Fenis A Demaria O Gauthier L Vivier E Narni-Mancinelli E . New immune cell engagers for cancer immunotherapy. Nat Rev Immunol. (2024) 24:471–86. doi: 10.1038/s41577-023-00982-7. PMID: 38273127

[42] Braster R O'Toole T van Egmond M . Myeloid cells as effector cells for monoclonal antibody therapy of cancer. Methods. (2014) 65:28–37. doi: 10.1016/j.ymeth.2013.06.020. PMID: 23811299

[43] Biglari A Southgate TD Fairbairn LJ Gilham DE . Human monocytes expressing a CEA-specific chimeric CD64 receptor specifically target CEA-expressing tumour cells *in vitro* and *in vivo*. Gene Ther. (2006) 13:602–10. doi: 10.1038/sj.gt.3302706. PMID: 16397508

[44] Shapir Itai Y Barboy O Salomon R Bercovich A Xie K Winter E . Bispecific dendritic-T cell engager potentiates anti-tumor immunity. Cell. (2024) 187:375–89. doi: 10.1016/j.cell.2023.12.011. PMID: 38242085

[45] Leclercq-Cohen G Steinhoff N Alberti Servera L Nassiri S Danilin S Piccione E . Dissecting the mechanisms underlying the cytokine release syndrome (CRS) mediated by T-cell bispecific antibodies. Clin Cancer Res. (2023) 29:4449–63. doi: 10.1158/1078-0432.ccr-22-3667. PMID: 37379429 PMC10618647

[46] Morris EC Neelapu SS Giavridis T Sadelain M . Cytokine release syndrome and associated neurotoxicity in cancer immunotherapy. Nat Rev Immunol. (2022) 22:85–96. doi: 10.1038/s41577-021-00547-6. PMID: 34002066 PMC8127450

[47] Sabari JK Lok BH Laird JH Poirier JT Rudin CM . Unravelling the biology of SCLC: implications for therapy. Nat Rev Clin Oncol. (2017) 14:549–61. doi: 10.1038/nrclinonc.2017.71. PMID: 28534531 PMC5843484

[48] Saunders LR Bankovich AJ Anderson WC Aujay MA Bheddah S Black K . A DLL3-targeted antibody-drug conjugate eradicates high-grade pulmonary neuroendocrine tumor-initiating cells *in vivo*. Sci Transl Med. (2015) 7:302ra136. doi: 10.1126/scitranslmed.aac9459. PMID: 26311731 PMC4934375

[49] Giffin MJ Cooke K Lobenhofer EK Estrada J Zhan J Deegen P . AMG 757, a half-life extended, DLL3-targeted bispecific T-cell engager, shows high potency and sensitivity in preclinical models of small-cell lung cancer. Clin Cancer Res. (2021) 27:1526–37. doi: 10.1158/1078-0432.ccr-20-2845. PMID: 33203642

[50] Owen DH Giffin MJ Bailis JM Smit MD Carbone DP He K . DLL3: an emerging target in small cell lung cancer. J Hematol Oncol. (2019) 12:61. doi: 10.1186/s13045-019-0745-2. PMID: 31215500 PMC6582566

[51] Dhillon S . Tarlatamab: first approval. Drugs. (2024) 84:995–1003. doi: 10.1007/s40265-024-02070-z. PMID: 39023700

[52] Mountzios G Sun L Cho BC Demirci U Baka S Gumus M . Tarlatamab in small-cell lung cancer after platinum-based chemotherapy. N Engl J Med. (2025) 393:349–61. doi: 10.1056/nejmoa2502099. PMID: 40454646

[53] Hipp S Voynov V Drobits-Handl B Giragossian C Trapani F Nixon AE . A bispecific DLL3/CD3 IgG-like T-cell engaging antibody induces antitumor responses in small cell lung cancer. Clin Cancer Res. (2020) 26:5258–68. doi: 10.1158/1078-0432.ccr-20-0926. PMID: 32554516

[54] Wermke M Gambardella V Kuboki Y Felip E Sanmamed MF Alese OB . Phase I dose-escalation results for the delta-like ligand 3/CD3 IgG-like T-cell engager obrixtamig (BI 764532) in patients with delta-like ligand 3+ small cell lung cancer or neuroendocrine carcinomas. J Clin Oncol. (2025) 43:3021–31. doi: 10.1200/jco-25-00363. PMID: 40706016 PMC12440289

[55] Wermke M Alt J Bozorgmehr F Fuchs F Rusquec PD Gazzah A . DAREONTM-9, a phase Ib study of obrixtamig plus topotecan in patients (pts) with advanced small cell lung cancer (SCLC): Interim analysis results. J Clin Oncol. (2025) 43:8094. doi: 10.1200/jco.2025.43.16_suppl.8094. PMID: 40980778

[56] Herbst RS . Review of epidermal growth factor receptor biology. Int J Radiat Oncol Biol Phys. (2004) 59:21–6. doi: 10.1016/j.ijrobp.2003.11.041. PMID: 15142631

[57] Scagliotti GV Selvaggi G Novello S Hirsch FR . The biology of epidermal growth factor receptor in lung cancer. Clin Cancer Res. (2004) 10:4227s–32s. doi: 10.1158/1078-0432.ccr-040007. PMID: 15217963

[58] Wingert S Reusch U Knackmuss S Kluge M Damrat M Pahl J . Preclinical evaluation of AFM24, a novel CD16A-specific innate immune cell engager targeting EGFR-positive tumors. MAbs. (2021) 13:1950264. doi: 10.1080/19420862.2021.1950264. PMID: 34325617 PMC8331026

[59] El-Khoueiry A Saavedra O Thomas J Livings C Garralda E Hintzen G . First-in-human phase I study of a CD16A bispecific innate cell engager, AFM24, targeting EGFR-expressing solid tumors. Clin Cancer Res. (2025) 31:1257–67. doi: 10.1158/1078-0432.ccr-24-1991. PMID: 39846810 PMC11964176

[60] Kim HR Oberoi A Lopez JS El-Khoueiry AB Saavedra O Thomas JS . Bispecific innate cell engager (ICE) AFM24 in combination with atezolizumab in patients with advanced/metastatic EGFR-expressing non-small cell lung cancer (NSCLC) without driver mutations: Initial results from a phase 2a study. J Clin Oncol. (2025) 43:2609. doi: 10.1200/jco.2025.43.16_suppl.2609

[61] Aaron WH Austin R Barath M Callihan E Cremin M Evans T . Abstract C033: HPN328: An anti-DLL3 T cell engager for treatment of small cell lung cancer. Mol Cancer Ther. (2019) 18:C033–3. doi: 10.1158/1535-7163.targ-19-c033. PMID: 41680580

[62] Austin RJ Lemon BD Aaron WH Barath M Culp PA DuBridge RB . TriTACs, a novel class of T-cell-engaging protein constructs designed for the treatment of solid tumors. Mol Cancer Ther. (2021) 20:109–20. doi: 10.1158/1535-7163.mct-20-0061. PMID: 33203731

[63] Molloy ME Austin RJ Lemon BD Aaron WH Ganti V Jones A . Preclinical characterization of HPN536, a trispecific, T-cell-activating protein construct for the treatment of mesothelin-expressing solid tumors. Clin Cancer Res. (2021) 27:1452–62. doi: 10.1158/1078-0432.ccr-20-3392. PMID: 33262134

[64] Molloy ME Aaron WH Barath M Bush MC Callihan EC Carlin K . HPN328, a trispecific T cell-activating protein construct targeting DLL3-expressing solid tumors. Mol Cancer Ther. (2024) 23:1294–304. doi: 10.1158/1535-7163.mct-23-0524. PMID: 38670552 PMC11372363

[65] Beltran H Johnson ML Jain P Schenk EL Sanborn RE Thompson JR . Updated results from a phase 1/2 study of HPN328, a tri-specific, half-life (T1/2) extended DLL3-targeting T-cell engager in patients (pts) with small cell lung cancer (SCLC) and other neuroendocrine cancers (NEC). J Clin Oncol. (2024) 42:8090. doi: 10.1200/jco.2024.42.16_suppl.8090. PMID: 41735675

[66] Wang Q Shi J Chai X Zheng L Wu L Mou H . A phase 1 dose escalation and expansion study of ZG006, a trispecific T cell engager targeting CD3/DLL3/DLL3, as monotherapy in patients with refractory small cell lung cancer or neuroendocrine carcinoma. J Clin Oncol. (2025) 43:8089. doi: 10.1200/jco.2025.43.16_suppl.8089. PMID: 40980778

[67] Ai X Zhang YY Zhang T Yi T Li M Yao W . A phase 2 dose expansion study of ZG006, a trispecific T cell engager targeting CD3/DLL3/DLL3, as monotherapy in patients with advanced small cell lung cancer. J Clin Oncol. (2025) 43:8007. doi: 10.1200/jco.2025.43.16_suppl.8007. PMID: 40980778

[68] Yonezawa A Dutt S Chester C Kim J Kohrt HE . Boosting cancer immunotherapy with anti-CD137 antibody therapy. Clin Cancer Res. (2015) 21:3113–20. doi: 10.1158/1078-0432.ccr-15-0263. PMID: 25908780 PMC5422104

[69] Liu G Luo P . Targeting CD137 (4-1BB) towards improved safety and efficacy for cancer immunotherapy. Front Immunol. (2023) 14:1208788. doi: 10.3389/fimmu.2023.1208788. PMID: 37334375 PMC10272836

[70] Chiu D Tavare R Haber L Aina OH Vazzana K Ram P . A PSMA-targeting CD3 bispecific antibody induces antitumor responses that are enhanced by 4-1BB costimulation. Cancer Immunol Res. (2020) 8:596–608. doi: 10.1158/2326-6066.cir-19-0518. PMID: 32184296

[71] Claus C Ferrara C Xu W Sam J Lang S Uhlenbrock F . Tumor-targeted 4-1BB agonists for combination with T cell bispecific antibodies as off-the-shelf therapy. Sci Transl Med. (2019) 11:eaav5989. doi: 10.1126/scitranslmed.aav5989. PMID: 31189721 PMC7181714

[72] Mikami H Feng S Matsuda Y Ishii S Naoi S Azuma Y . Engineering CD3/CD137 dual specificity into a DLL3-targeted T-cell engager enhances T-cell infiltration and efficacy against small-cell lung cancer. Cancer Immunol Res. (2024) 12:719–30. doi: 10.1158/2326-6066.cir-23-0638. PMID: 38558120

[73] Huang SW Pan CM Lin YC Chen MC Chen Y Jan CI . BiTE-secreting CAR-gammadeltaT as a dual targeting strategy for the treatment of solid tumors. Adv Sci (Weinh). (2023) 10:e2206856. doi: 10.1002/advs.202206856. PMID: 37078788 PMC10265101

[74] Chen MC Hung MY Pan CM Huang SW Jan CI Li YH . Pemetrexed combined with dual immune checkpoint blockade enhances cytotoxic T lymphocytes against lung cancer. Cancer Sci. (2023) 114:2761–73. doi: 10.1111/cas.15806. PMID: 37017116 PMC10323078

[75] Lin YC Chen MC Huang SW Chen Y Ho JH Lin FY . Targeting dual immune checkpoints PD-L1 and HLA-G by trispecific T cell engager for treating heterogeneous lung cancer. Adv Sci (Weinh). (2024) 11:e2309697. doi: 10.1002/advs.202309697. PMID: 39234811 PMC11538689

[76] Herrera M Pretelli G Desai J Garralda E Siu LL Steiner TM . Bispecific antibodies: advancing precision oncology. Trends Cancer. (2024) 10:893–919. doi: 10.1016/j.trecan.2024.07.002. PMID: 39214782

[77] Pan H Liu J Deng W Xing J Li Q Wang Z . Site-specific PEGylation of an anti-CEA/CD3 bispecific antibody improves its antitumor efficacy. Int J Nanomedicine. (2018) 13:3189–201. doi: 10.2147/ijn.s164542. PMID: 29881272 PMC5985803

[78] Tijink BM Laeremans T Budde M Stigter-van Walsum M Dreier T de Haard HJ . Improved tumor targeting of anti-epidermal growth factor receptor nanobodies through albumin binding: taking advantage of modular nanobody technology. Mol Cancer Ther. (2008) 7:2288–97. doi: 10.1158/1535-7163.mct-07-2384. PMID: 18723476

[79] Mandrup OA Ong SC Lykkemark S Dinesen A Rudnik-Jansen I Dagnaes-Hansen NF . Programmable half-life and anti-tumour effects of bispecific T-cell engager-albumin fusions with tuned FcRn affinity. Commun Biol. (2021) 4:310. doi: 10.1038/s42003-021-01790-2. PMID: 33686177 PMC7940400

[80] Lorenczewski G Friedrich M Kischel R Dahlhoff C Anlahr J Balazs M . Generation of a half-life extended anti-CD19 BiTE® antibody construct compatible with once-weekly dosing for treatment of CD19-positive Malignancies. Blood. (2017) 130:2815. doi: 10.1182/blood.V130.Suppl_1.2815.2815, PMID: 41761659

[81] Dingman R Balu-Iyer SV . Immunogenicity of protein pharmaceuticals. J Pharm Sci. (2019) 108:1637–54. doi: 10.1016/j.xphs.2018.12.014. PMID: 30599169 PMC6720129

[82] Middelburg J Kemper K Engelberts P Labrijn AF Schuurman J van Hall T . Overcoming challenges for CD3-bispecific antibody therapy in solid tumors. Cancers (Basel). (2021) 13:287. doi: 10.3390/cancers13020287. PMID: 33466732 PMC7829968

[83] Kebenko M Goebeler ME Wolf M Hasenburg A Seggewiss-Bernhardt R Ritter B . A multicenter phase 1 study of solitomab (MT110, AMG 110), a bispecific EpCAM/CD3 T-cell engager (BiTE(R)) antibody construct, in patients with refractory solid tumors. Oncoimmunology. (2018) 7:e1450710. doi: 10.1080/2162402x.2018.1450710. PMID: 30221040 PMC6136859

[84] Borlak J Langer F Spanel R Schondorfer G Dittrich C . Immune-mediated liver injury of the cancer therapeutic antibody catumaxomab targeting EpCAM, CD3 and Fcgamma receptors. Oncotarget. (2016) 7:28059–74. doi: 10.18632/oncotarget.8574. PMID: 27058902 PMC5053709

[85] Grunnet M Sorensen JB . Carcinoembryonic antigen (CEA) as tumor marker in lung cancer. Lung Cancer. (2012) 76:138–43. doi: 10.1016/j.lungcan.2011.11.012. PMID: 22153832

[86] Wei X Lai Y Li J Qin L Xu Y Zhao R . PSCA and MUC1 in non-small-cell lung cancer as targets of chimeric antigen receptor T cells. Oncoimmunology. (2017) 6:e1284722. doi: 10.1080/2162402x.2017.1284722. PMID: 28405515 PMC5384358

[87] Zhang H Yang Y Li X Yuan X Chu Q . Targeting the Notch signaling pathway and the Notch ligand, DLL3, in small cell lung cancer. BioMed Pharmacother. (2023) 159:114248. doi: 10.1016/j.biopha.2023.114248. PMID: 36645960

[88] Schmidt LH Heitkotter B Schulze AB Schliemann C Steinestel K Trautmann M . Prostate specific membrane antigen (PSMA) expression in non-small cell lung cancer. PloS One. (2017) 12:e0186280. doi: 10.1371/journal.pone.0186280. PMID: 29077706 PMC5659610

[89] Bo Q Zhang MG Yang F Zheng Y Li ZL Zheng YM . Somatostatin receptor 2 targeting peptide modifications for peptide-drug conjugate treatment of small cell lung cancer. Acta Pharmacol Sin. (2025) 46(12):3291–3301. doi: 10.1038/s41401-025-01584-w. PMID: 40533489 PMC12644740

[90] Susac L Vuong MT Thomas C von Bulow S O'Brien-Ball C Santos AM . Structure of a fully assembled tumor-specific T cell receptor ligated by pMHC. Cell. (2022) 185:3201–13:e3219. doi: 10.1016/j.cell.2022.07.010, PMID: 35985289 PMC9630439

[91] Kaczorowski M Chlopek M Kruczak A Rys J Lasota J Miettinen M . PRAME expression in cancer. A systematic immunohistochemical study of >5800 epithelial and nonepithelial tumors. Am J Surg Pathol. (2022) 46:1467–76. doi: 10.1097/pas.0000000000001944. PMID: 35973038 PMC9588667

[92] Bacac M Fauti T Sam J Colombetti S Weinzierl T Ouaret D . A novel carcinoembryonic antigen T-cell bispecific antibody (CEA TCB) for the treatment of solid tumors. Clin Cancer Res. (2016) 22:3286–97. doi: 10.1158/1078-0432.ccr-15-1696. PMID: 26861458

[93] Segal NH Melero I Moreno V Steeghs N Marabelle A Rohrberg K . CEA-CD3 bispecific antibody cibisatamab with or without atezolizumab in patients with CEA-positive solid tumours: results of two multi-institutional phase 1 trials. Nat Commun. (2024) 15:4091. doi: 10.1038/s41467-024-48479-8. PMID: 38750034 PMC11096172

[94] Correnti CE Laszlo GS de van der Schueren WJ Godwin CD Bandaranayake A Busch MA . Simultaneous multiple interaction T-cell engaging (SMITE) bispecific antibodies overcome bispecific T-cell engager (BiTE) resistance via CD28 co-stimulation. Leukemia. (2018) 32:1239–43. doi: 10.1038/s41375-018-0014-3. PMID: 29588544 PMC5943151

[95] Piha-Paul S Olwill SA Hamilton E Tolcher A Pohlmann P Liu SV . A first-in-human study of cinrebafusp alfa, a HER2/4-1BB bispecific molecule, in patients with HER2-positive advanced solid Malignancies. Clin Cancer Res. (2025) 31:288–98. doi: 10.1158/1078-0432.ccr-24-1552. PMID: 39235868 PMC11739778

[96] Krupka C Kufer P Kischel R Zugmaier G Lichtenegger FS Kohnke T . Blockade of the PD-1/PD-L1 axis augments lysis of AML cells by the CD33/CD3 BiTE antibody construct AMG 330: reversing a T-cell-induced immune escape mechanism. Leukemia. (2016) 30:484–91. doi: 10.1038/leu.2015.214. PMID: 26239198

[97] Chen X Amar N Zhu Y Wang C Xia C Yang X . Combined DLL3-targeted bispecific antibody with PD-1 inhibition is efficient to suppress small cell lung cancer growth. J Immunother Cancer. (2020) 8:e000785. doi: 10.1136/jitc-2020-000785. PMID: 32554616 PMC7304844

[98] Paulson KG Lau SCM Ahn MJ Moskovitz M Pogorzelski M Hafliger S . Safety and activity of tarlatamab in combination with a PD-L1 inhibitor as first-line maintenance therapy after chemo-immunotherapy in patients with extensive-stage small-cell lung cancer (DeLLphi-303): a multicentre, non-randomised, phase 1b study. Lancet Oncol. (2025) 26:1300–11. doi: 10.1016/s1470-2045(25)00480-2. PMID: 40934933

[99] Zhao S Zhao H Yang W Zhang L . The next generation of immunotherapies for lung cancers. Nat Rev Clin Oncol. (2025) 22:592–616. doi: 10.1038/s41571-025-01035-9. PMID: 40528044

[100] Goebeler ME Stuhler G Bargou R . Bispecific and multispecific antibodies in oncology: opportunities and challenges. Nat Rev Clin Oncol. (2024) 21:539–60. doi: 10.1038/s41571-024-00905-y. PMID: 38822215

[101] Ma H Wang H Sove RJ Wang J Giragossian C Popel AS . Combination therapy with T cell engager and PD-L1 blockade enhances the antitumor potency of T cells as predicted by a QSP model. J Immunother Cancer. (2020) 8:e001141. doi: 10.1136/jitc-2020-001141. PMID: 32859743 PMC7454244

[102] Fan Y Duan Y Chen J Wang Y Shang K Jiang J . Bispecific killer cell engager-secreting CAR-T cells redirect natural killer specificity to enhance antitumour responses. Nat BioMed Eng. (2025) 10(2):390–403. doi: 10.1038/s41551-025-01450-4. PMID: 40659834

[103] Yang Z Cheng C Li Z Wang H Zhang M Xie E . Advancing liver cancer treatment with dual-targeting CAR-T therapy. J Nanobiotechnology. (2025) 23:462. doi: 10.1186/s12951-025-03512-w. PMID: 40551157 PMC12186358

